# Metabolic dysfunction–associated steatotic liver disease and heart failure with preserved ejection fraction: mechanisms and clinical implications from a heart–liver metabolic axis perspective

**DOI:** 10.3389/fphar.2026.1863710

**Published:** 2026-05-28

**Authors:** Xuebi Wu, Yongxin Ma, Xueshi Yin, Jianping Liu, Yongheng Zhang

**Affiliations:** 1 Department of Clinical Medicine, North Sichuan Medical College, Nanchong, Sichuan, China; 2 Department of Cardiac and Great Vascular Surgery, Suining Central Hospital, Suining, Sichuan, China

**Keywords:** clinical management, heart failure with preserved ejection fraction, heart–liver metabolic axis, insulin resistance, metabolic dysfunction–associated steatotic liver disease, risk stratification

## Abstract

Metabolic dysfunction–associated steatotic liver disease (MASLD) and heart failure with preserved ejection fraction (HFpEF) are increasingly prevalent cardiometabolic disorders that frequently coexist in clinical practice. Growing evidence indicates that MASLD is associated with an increased risk of heart failure, with emerging data suggesting particular relevance to the HFpEF phenotype. This relationship is supported by several shared pathophysiological mechanisms, including insulin resistance, lipotoxicity, chronic low-grade inflammation, neurohormonal activation, microvascular dysfunction, fibrotic remodeling, and dysregulation of the gut–heart–liver axis, collectively supporting the concept of a heart–liver metabolic axis linking hepatic and cardiac injury. Clinically, MASLD, especially when accompanied by a greater fibrosis burden, may help identify a metabolically enriched HFpEF subgroup characterized by more advanced systemic derangement and organ remodeling. Although lifestyle intervention, sodium–glucose cotransporter 2 (SGLT2) inhibitors, incretin-based therapies, and liver-directed treatments have broadened the therapeutic framework for this overlap phenotype, prospective evidence specifically addressing patients with coexisting MASLD and HFpEF remains limited. This review summarizes the epidemiological links, shared mechanisms, risk stratification approaches, and therapeutic strategies relevant to the coexistence of MASLD and HFpEF.

## 1 Introduction

Metabolic dysfunction–associated steatotic liver disease (MASLD) has emerged as one of the most prevalent chronic liver disorders worldwide and is an important cause of cirrhosis and other liver-related complications (Toh et al., 2022). As an updated nomenclature replacing the former framework of nonalcoholic fatty liver disease (NAFLD), MASLD places greater emphasis on the central role of metabolic dysfunction in disease development and highlights its close association with cardiometabolic risk factors such as obesity, type 2 diabetes mellitus, hypertension, and dyslipidemia (Eslam et al., 2020).

Heart failure with preserved ejection fraction (HFpEF) is increasingly recognized as a major clinical phenotype of heart failure. Its development and progression are closely linked to obesity, insulin resistance, chronic low-grade inflammation, and adipose tissue dysfunction (Steinberg et al., 2012; Tsao et al., 2018; Peikert et al., 2025; Schiattarella et al., 2021). Compared with other heart failure phenotypes, HFpEF is characterized by greater clinical heterogeneity and a more complex multiorgan metabolic background, making early identification and risk stratification particularly challenging.

These observations support consideration of MASLD and HFpEF within a shared cardiometabolic framework. A recent nationwide Korean cohort study including nearly 9 million adults showed that MASLD, as defined by the updated criteria, was independently associated with a 38% higher risk of incident heart failure compared with the absence of steatotic liver disease (HR 1.38, 95% CI 1.35–1.41), and further suggested that this association may be more pronounced in individuals with a greater fibrosis burden (Lee et al., 2024; Younossi et al., 2023). MASLD and HFpEF also appear to interact through shared mechanisms involving insulin resistance, lipotoxicity, neurohormonal activation, chronic low-grade inflammation, endothelial dysfunction, and dysregulation of the gut–heart–liver axis (Lee et al., 2024; Zhou et al., 2025; Mantovani et al., 2021; Zhou et al., 2023). However, most existing studies have examined MASLD and HFpEF either in parallel or in isolation, and have focused mainly on selected epidemiological associations or individual mechanistic pathways rather than on their bidirectional interaction within a heart–liver metabolic axis framework. Although the association between MASLD and atherosclerotic cardiovascular disease is well established, its specific implications for the development, phenotypic stratification, and clinical management of HFpEF remain insufficiently defined (Duell et al., 2022; Chang et al., 2025). This review therefore provides an integrated discussion of the epidemiological links, shared pathophysiological basis, risk stratification, and therapeutic implications of MASLD–HFpEF overlap, with particular emphasis on their bidirectional interaction within the heart–liver metabolic axis.

Most studies examining the relationship between steatotic liver disease and heart failure were conducted under the earlier NAFLD nomenclature. Given the substantial overlap between historical NAFLD populations and those currently classified as MASLD, as well as their broadly similar clinical characteristics and outcomes, we retain the original study definitions when citing individual studies while primarily using the term MASLD throughout this review (Hagström et al., 2024; Hashida et al., 2024; Younossi et al., 2024).

## 2 MASLD and HFpEF

MASLD represents a disease spectrum that extends from simple steatosis to metabolic dysfunction–associated steatohepatitis (MASH), fibrosis, cirrhosis, and hepatic decompensation (Rinella et al., 2023a). Within this spectrum, MASH is characterized by active inflammation and a greater propensity for progressive fibrosis (Le et al., 2023; Younossi et al., 2018). This progression is driven by lipotoxicity and oxidative stress, which induce hepatocellular injury, trigger the release of damage-associated molecular patterns (DAMPs), and activate Kupffer cells, thereby amplifying inflammatory signaling and allowing liver injury to evolve into a broader state of systemic metabolic inflammation (Iturbe-Rey et al., 2025).

**FIGURE 1:**
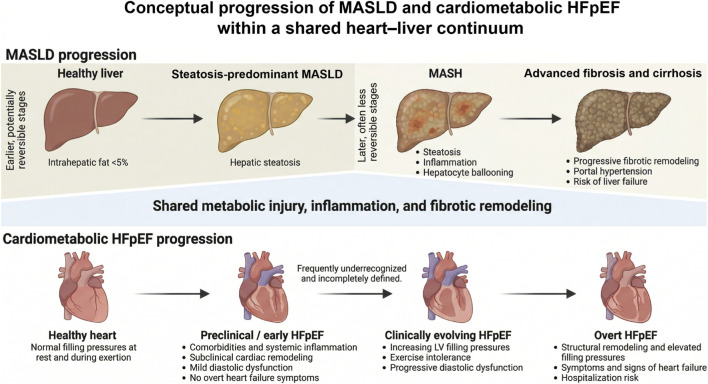
Conceptual progression of MASLD and cardiometabolic HFpEF within a shared heart–liver–metabolic continuum. This figure illustrates the parallel progression of metabolic dysfunction–associated steatotic liver disease (MASLD) and cardiometabolic heart failure with preserved ejection fraction (HFpEF) within a shared heart–liver continuum. In the upper panel, liver disease progresses from a healthy liver to steatosis-predominant MASLD, metabolic dysfunction–associated steatohepatitis (MASH), and advanced fibrosis/cirrhosis, with increasing steatosis, inflammation, hepatocyte injury, and fibrotic remodeling and with decreasing reversibility across stages. In the lower panel, cardiometabolic HFpEF progresses from a healthy heart to preclinical/early HFpEF, clinically evolving HFpEF, and overt HFpEF, accompanied by systemic inflammation, subclinical cardiac remodeling, progressive diastolic dysfunction, rising filling pressures, and overt heart failure manifestations. The central band highlights shared metabolic injury, inflammation, and fibrotic remodeling as common processes linking hepatic disease progression and cardiometabolic HFpEF development. Abbreviations: MASLD, metabolic dysfunction–associated steatotic liver disease; HFpEF, heart failure with preserved ejection fraction; MASH, metabolic dysfunction–associated steatohepatitis.

Hepatic inflammation and fibrosis are associated not only with adverse liver-related outcomes and increased all-cause mortality, but also with abnormalities in cardiac structure and function (Younossi et al., 2016). Available evidence suggests that diastolic dysfunction correlates with the severity of liver fibrosis, whereas advanced liver disease may further impair cardiac function through mechanisms such as cirrhotic cardiomyopathy (Singh et al., 2015; Chung et al., 2018). These observations suggest that progression along the MASLD spectrum reflects broader disturbances in inter-organ crosstalk rather than local hepatic injury alone. Accordingly, increasing liver disease severity, particularly a greater fibrosis burden, may identify a subgroup with more advanced metabolic derangement and a higher likelihood of cardiac involvement. As illustrated in Figure 1, MASLD and cardiometabolic HFpEF may therefore be understood as interconnected manifestations of shared metabolic stress, inflammation, and fibrosis-related remodeling.

HFpEF is generally defined as a heart failure phenotype with preserved left ventricular ejection fraction (LVEF ≥50%), and its development and progression are closely linked to obesity, insulin resistance, chronic low-grade inflammation, abnormalities in energy metabolism, and multiorgan dysfunction. In this context, HFpEF is better understood as a syndrome that evolves gradually from metabolic stress and subclinical organ remodeling, rather than as an isolated abnormality of cardiac function.

The coexistence of MASLD and HFpEF is therefore better viewed as a higher-risk state characterized by greater metabolic inflammation and more pronounced inter-organ interaction than as a chance concurrence of two common disorders. Viewing these two conditions within a shared heart–liver–metabolic continuum may help clarify their parallel trajectories of progression and provide a conceptual framework for subsequent discussion of epidemiology, mechanisms, risk stratification, and treatment.

## 3 Epidemiological links between MASLD and HFpEF

### 3.1 Cardiometabolic burden of MASLD

MASLD is currently one of the most common chronic liver diseases worldwide, affecting approximately one-quarter of the adult population, and its prevalence continues to rise in parallel with increasing rates of obesity and type 2 diabetes mellitus, thereby imposing a substantial public health burden (Younossi et al., 2025a). Beyond cirrhosis and other adverse liver-related outcomes, MASLD is frequently accompanied by cardiometabolic comorbidities such as hypertension, obesity, type 2 diabetes, chronic kidney disease, and dyslipidemia, together creating a persistent background of metabolic disturbance and chronic inflammation. MASLD should therefore be viewed as part of a broader state of multiorgan cardiometabolic dysfunction, which provides an epidemiological basis for its marked overlap with HFpEF.

### 3.2 High coexistence of MASLD in HFpEF

Available evidence indicates that steatotic liver disease—historically reported mostly as NAFLD and largely overlapping with the current MASLD spectrum—is highly prevalent among patients with heart failure, with an even greater burden observed in those with HFpEF (Rinella et al., 2023a). Studies using imaging or noninvasive fibrosis assessment suggest that steatotic liver disease is common in HFpEF and may be more prevalent than in the general population, with prevalence estimates of 27% in a prospectively enrolled HFpEF cohort and 50% in the subgroup with prior abdominal imaging (Miller et al., 2020). Compared with HFrEF, HFpEF more commonly presents as a cardiometabolic syndrome driven by obesity, insulin resistance, and chronic low-grade inflammation (Schiattarella et al., 2021). The frequent coexistence of MASLD and HFpEF likely reflects a shared pathobiological substrate rather than simple coincidence.

Further studies have shown that patients with HFpEF and coexisting steatotic liver disease are more likely to exhibit an obesity-related phenotype, reduced exercise capacity, and more pronounced multiorgan metabolic abnormalities (Itier et al., 2021). These observations suggest that hepatic steatosis in HFpEF may represent an organ-level manifestation of the underlying metabolically inflammatory phenotype rather than an incidental accompanying finding (Kitzman and Nicklas, 2018). Clinically, the high prevalence of MASLD in HFpEF may help identify a subgroup with greater metabolic burden and a more pronounced inflammatory profile.

### 3.3 Longitudinal evidence linking MASLD to HFpEF-related events

Longitudinal studies further support an association between MASLD and an increased risk of incident heart failure beyond their cross-sectional coexistence. Representative studies and their main findings are summarized in Table 1.

**TABLE 1:** Longitudinal evidence linking MASLD or related hepatic abnormalities to heart failure risk, including HFpEF-relevant findings where available.

Study	Study design/Population	Hepatic exposure definition	HF outcome	Follow-up	Effect estimate	Key finding	Ref
Framingham Heart Study	Prospective community-based cohort; individuals without prior HF (n = 3, 544)	GGT as a surrogate marker of hepatic/metabolic dysfunction	Incident HF	∼24 years	HR 1.39 (95% CI 1.20–1.62) per 1-SD increase in log-transformed GGT	Higher GGT levels were associated with greater future HF risk, suggesting that hepatic/metabolic abnormalities may precede overt HF	Dhingra et al. (2010)
Medicare cohort	Retrospective population-based cohort of Medicare beneficiaries (n > 870, 000)	Clinically diagnosed NAFLD (historical nomenclature)	Incident HF, with subtype analysis	∼1 year	HR 1.23 (95% CI 1.18–1.29)	Clinically recognized fatty liver disease was independently associated with incident HF, with a stronger signal for HFpEF than for HFrEF	Fudim et al. (2021)
NHANES-based study	Nationally representative weighted sample of U. S. adults (>80 million weighted population)	Fatty Liver Index (FLI)	HF-related risk and adverse outcomes	∼19 years	aOR ≈3.5 for HF; all-cause mortality HR ≈ 1.9	FLI-defined fatty liver was associated with greater HF-related burden and adverse long-term outcomes, although exposure and outcome ascertainment were less specific than in imaging- or biopsy-based cohorts	Minhas et al. (2022)
Kailuan cohort	Population-based longitudinal cohort from China (n = 98, 685)	Ultrasound-defined fatty liver disease	Incident HF	∼14 years	HR 1.40 (95% CI 1.30–1.50)	Ultrasound-defined fatty liver disease was associated with increased HF risk, with a graded rise according to steatosis burden and fibrosis risk	Wei et al. (2023)
National Taiwan University Hospital cohort	Longitudinal cohort of patients with fatty liver disease (n = 26, 676)	Ultrasound-defined MASLD/fatty liver disease	Incident HF; ∼75% of cases were HFpEF	∼15 years	SHR 2.59	MASLD was strongly associated with incident HF, predominantly HFpEF, and risk increased with cumulative cardiometabolic burden	Chang et al. (2025)
Swedish nationwide biopsy-confirmed cohort	Nationwide matched cohort of biopsy-confirmed fatty liver disease (n = 10, 422)	Histologically confirmed fatty liver disease	Incident congestive HF	>13 years	aHR 1.75 (95% CI 1.63–1.87)	Histology-confirmed fatty liver disease was associated with substantially increased HF risk, which further increased with greater histologic severity	Simon et al. (2022)
Systematic review and meta-analysis	11 cohort studies including >11.24 million adults	NAFLD/MASLD defined variably across studies	Incident HF	Median ∼10 years	HR 1.50 (95% CI 1.34–1.67)	Across cohort studies, fatty liver disease showed a consistent association with incident HF after adjustment for conventional cardiometabolic risk factors	Mantovani et al. (2022)

Abbreviations: MASLD, metabolic dysfunction-associated steatotic liver disease; NAFLD, nonalcoholic fatty liver disease; GGT, gamma-glutamyl transferase; FLI, fatty liver index; HF, heart failure; HFpEF, heart failure with preserved ejection fraction; HFrEF, heart failure with reduced ejection fraction; HR, hazard ratio; aHR, adjusted hazard ratio; aOR, adjusted odds ratio; SHR, subdistribution hazard ratio.

Earlier studies used the historical NAFLD, nomenclature or surrogate markers of hepatic dysfunction; these original definitions are retained here to accurately reflect the source literature. If the HF, estimate in the NHANES-based study reflects prevalent rather than incident HF, this row may be more appropriately regarded as supportive population-based evidence rather than core longitudinal incident HF, evidence.

The earliest clues came from surrogate markers such as liver enzymes. In the Framingham Heart Study, elevated gamma-glutamyl transferase (GGT) levels were associated with an increased long-term risk of heart failure; specifically, each 1-standard deviation increase in log-transformed GGT was associated with an approximately 39% higher risk of incident heart failure (Dhingra et al., 2010). This finding suggests that liver-derived metabolic or inflammatory abnormalities may already be present before the clinical onset of heart failure. Large population-based database studies subsequently provided further support for this association. In a U. S. Medicare cohort, clinically diagnosed steatotic liver disease was independently associated with a 23% increase in the risk of incident heart failure, and subtype analyses suggested that this association was more pronounced for HFpEF than for HFrEF (29). Similarly, a study based on a weighted NHANES sample found that steatotic liver disease defined by the Fatty Liver Index was associated with a higher burden of heart failure and increased all-cause mortality (Minhas et al., 2022; Bedogni et al., 2006; Ruhl and Everhart, 2015). Although exposure and outcome ascertainment in these studies was less specific, the consistency of direction across large samples remains informative.

Imaging-based studies have provided more direct support for this relationship. In the Kailuan cohort from China, ultrasound-defined steatotic liver disease was associated with an approximately 40% higher risk of incident heart failure, and this risk increased progressively with greater severity of hepatic steatosis and fibrosis (Wei et al., 2023). A longitudinal study from National Taiwan University Hospital further showed that ultrasound-defined MASLD was significantly associated with incident heart failure, with approximately three-quarters of new heart failure events classified as HFpEF. In addition, the risk of heart failure increased in parallel with the accumulation of cardiometabolic risk factors (Chang et al., 2025). Together, these studies indicate that the link between MASLD and HFpEF is reflected not only in an increased risk of heart failure, but also in the burden of liver disease and the cumulative degree of metabolic dysregulation.

Biopsy-confirmed cohorts further strengthen this association. In a Swedish nationwide cohort, patients with biopsy-confirmed steatotic liver disease had a markedly increased long-term risk of incident congestive heart failure, with an approximately 75% higher risk compared with controls, and this risk rose further with increasing histological severity of liver disease (Simon et al., 2022). These results extend observations from noninvasive studies and further support the view that liver fibrosis burden may reflect greater systemic metabolic and inflammatory stress.

Pooled analyses show a similar overall pattern. A systematic review including 11 cohort studies and more than 11 million adults reported an approximately 50% increase in incident heart failure risk associated with steatotic liver disease, and the association remained robust after adjustment for obesity, diabetes, and hypertension (Mantovani et al., 2022).

## 4 Shared pathophysiological mechanisms: the heart–liver axis

**FIGURE 2:**
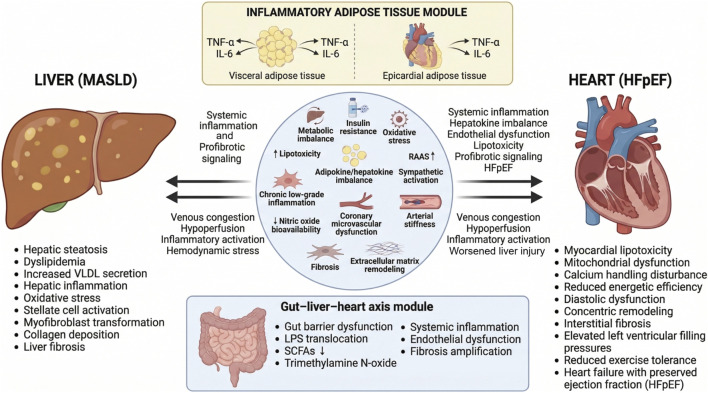
Shared mechanisms linking MASLD and HFpEF through the heart–liver axis. This figure summarizes the bidirectional interactions between metabolic dysfunction–associated steatotic liver disease (MASLD) and heart failure with preserved ejection fraction (HFpEF). The left panel shows major hepatic abnormalities in MASLD, and the right panel shows key cardiac abnormalities in HFpEF. The central module highlights shared mechanisms, including metabolic imbalance, insulin resistance, oxidative stress, lipotoxicity, chronic low-grade inflammation, endothelial and microvascular dysfunction, neurohormonal activation, arterial stiffness, and extracellular matrix remodeling. The upper and lower modules further illustrate the contributions of adipose tissue inflammation and the gut–liver–heart axis to fibrosis amplification and reciprocal liver–heart injury. Abbreviations: MASLD, metabolic dysfunction–associated steatotic liver disease; HFpEF, heart failure with preserved ejection fraction; VLDL, very-low-density lipoprotein; TNF-α, tumor necrosis factor-α; IL-6, interleukin-6; RAAS, renin–angiotensin–aldosterone system; LPS, lipopolysaccharide; SCFAs, short-chain fatty acids.

The link between MASLD and HFpEF is driven by multiple interconnected mechanisms, including metabolic imbalance, chronic inflammation, neurohormonal activation, microvascular dysfunction, and fibrotic remodeling, rather than by shared risk factors alone. Together, these bidirectional interactions between the liver and the heart support the view that MASLD–HFpEF represents a multiorgan metabolically inflammatory syndrome. The core pathophysiological framework is shown in Figure 2.

### 4.1 Metabolic imbalance and Lipotoxicity

Insulin resistance is a central metabolic mechanism linking MASLD and HFpEF. In the liver, impaired insulin signaling promotes lipogenesis and disrupts the normal regulation of gluconeogenesis and lipid metabolism, leading to hepatocellular lipid accumulation, dyslipidemia, and increased very-low-density lipoprotein (VLDL) secretion (Anstee et al., 2013; Sanders and Griffin, 2016; Ormazabal et al., 2018). These alterations extend beyond the liver and reflect a broader state of systemic metabolic remodeling.

Insulin resistance is also associated with enhanced adipose tissue lipolysis and elevated circulating free fatty acids (Badmus et al., 2023). Excess lipid delivery to the myocardium can induce lipotoxic injury, mitochondrial dysfunction, and disturbances in calcium homeostasis, thereby promoting interstitial remodeling and impaired diastolic compliance (Shimizu et al., 2010; Sletten et al., 2018). Insulin signaling normally helps maintain balanced myocardial substrate utilization (Abel, 2021). In insulin-resistant states, myocardial glucose uptake and oxidation decline, forcing the heart to rely more heavily on fatty acid oxidation (De Jong and Lopaschuk, 2017; Boudina et al., 2009; Karwi et al., 2018). Over time, this metabolic shift reduces energetic efficiency, increases oxidative stress, and promotes myocardial stiffness (Boudina et al., 2009; Kolwicz et al., 2013). In addition, impairment of the PI3K/Akt pathway and reduced nitric oxide bioavailability may further damage the microvascular endothelium and accelerate cardiac remodeling (Muniyappa and Sowers, 2013; Wende et al., 2015; Paulus and Tschöpe, 2013). Thus, insulin resistance provides a key mechanistic link between MASLD and HFpEF through lipotoxicity, altered energy metabolism, and microvascular injury.

### 4.2 Chronic low-grade inflammation and adipokine imbalance

Unlike heart failure syndromes driven predominantly by volume overload, HFpEF more commonly develops in the setting of obesity, diabetes, and related metabolic disturbances characterized by persistent low-grade inflammation (Schiattarella et al., 2021; Strocchi et al., 2024). In this context, visceral adipose tissue is not merely a passive energy reservoir, but an important amplifier of inflammatory signaling. Adipocyte hypertrophy is commonly accompanied by local hypoxia, cellular stress, and inflammatory cell infiltration, which promote the release of pro-inflammatory mediators such as TNF-α and IL-6 (Fujisaka et al., 2013; Gómez-Ambrosi, 2023).

In MASLD, visceral adipose dysfunction and hepatic inflammation reinforce one another, forming a persistent adipose–liver inflammatory loop (Zhao et al., 2024; Burger et al., 2023). Increased oxidative stress worsens insulin resistance and may also disturb coagulation and fibrinolytic balance, thereby further amplifying the risks of atherosclerosis and heart failure (Uchida et al., 2012). Epicardial adipose tissue, because of its close proximity to the myocardium and coronary microvasculature, may further influence the myocardial interstitium and microvascular environment through paracrine signaling (Iozzo, 2011). Together, these tissues contribute to the shared metabolically inflammatory milieu of MASLD and HFpEF, thereby promoting diastolic dysfunction and cardiac remodeling (Strocchi et al., 2024).

### 4.3 Neurohormonal activation and vascular dysfunction

Persistent activation of the renin–angiotensin–aldosterone system (RAAS) and the sympathetic nervous system represents another shared amplifying mechanism in MASLD and HFpEF (Driessen et al., 2025). In the setting of metabolic dysfunction, RAAS upregulation promotes vasoconstriction, sodium and water retention, and elevated blood pressure, thereby increasing cardiac preload and afterload (Andreozzi et al., 2004; Mkhize et al., 2022; Yang et al., 2025; Kang et al., 2025). MASLD and heart failure also show substantial mechanistic overlap at the level of sympathetic regulation (Carnagarin et al., 2021). Chronic sympathetic overactivation is a hallmark of the metabolic syndrome and may further aggravate metabolic dysfunction and drive MASLD progression by increasing hepatic glucose output (Alvarez et al., 2002), suppressing insulin secretion (Schlaich et al., 2015), reducing peripheral glucose uptake, and promoting lipolysis (Lambert et al., 2010; Kawano and Cohen, 2013; Geerling et al., 2014).

Endothelial dysfunction provides an important mechanistic link across these processes (Targher et al., 2020). Metabolic disturbance and oxidative stress can suppress endothelial nitric oxide synthase (eNOS) activity and reduce nitric oxide bioavailability, thereby promoting vasoconstriction, inflammatory activation, and abnormal microvascular perfusion (Persico et al., 2017; Zhao et al., 2020). Inflammatory mediators such as TNF-α and IL-6 may further exacerbate endothelial inflammation through the NF-κB and MAPK signaling pathways, accelerating microvascular injury (Brasier, 2010; Ionescu et al., 2025). Against this background, arterial stiffness and hypertension increase left ventricular afterload, promote concentric remodeling, and reduce ventricular compliance. Impaired ventricular–arterial coupling also limits the ability of the heart to meet oxygen demand during exercise, thereby contributing to reduced exercise tolerance (Haykowsky et al., 2011; Chahal et al., 2010; Hu et al., 2024; Messerli et al., 2017). In HFpEF, this metabolically inflammatory state of microvascular and hemodynamic imbalance is a key driver of diastolic dysfunction and functional limitation.

### 4.4 Fibrosis and organ remodeling

Within the MASLD–HFpEF heart–liver axis, fibrosis is not merely a localized lesion confined to a single organ, but a shared structural consequence of persistent metabolic stress and inflammatory imbalance. Although the liver and heart differ in function, both organs may develop microvascular endothelial injury, activation of innate immunity, and excessive extracellular matrix (ECM) deposition under chronic injury conditions, ultimately leading to reduced tissue compliance and organ stiffening (Salah et al., 2021). Fibrosis can therefore be viewed as an important common end pathway linking MASLD progression to the HFpEF phenotype.

In the liver, activation of hepatic stellate cells (HSCs) is central to fibrogenesis. Lipotoxicity, oxidative stress, and inflammatory mediators promote their transition to a myofibroblast-like phenotype and stimulate collagen and other ECM deposition through signaling pathways such as TGF-β/Smad (Dewidar et al., 2019). Cytokines released by pro-inflammatory macrophages and injured hepatocytes further amplify inflammatory and profibrotic responses, while imbalance between ECM deposition and degradation drives progressive fibrosis (Pejnovic et al., 2016; Roeb, 2018; Zhu and Cai, 2025; Zhao et al., 2022).

In the heart, HFpEF is likewise characterized by interstitial remodeling and impaired diastolic compliance. Inflammation of the coronary microvascular endothelium reduces NO–cGMP–PKG signaling, thereby promoting fibroblast activation and collagen deposition (Paulus and Tschöpe, 2013). As interstitial collagen accumulates, matrix cross-linking increases and capillary density declines, leading to greater passive myocardial stiffness and lower left ventricular compliance, which together provide the structural basis for diastolic dysfunction (Borlaug et al., 2023).

Clinical studies increasingly suggest that liver fibrosis burden is closely associated with structural and functional abnormalities relevant to HFpEF. Greater fibrosis severity has been linked to more pronounced diastolic dysfunction, left atrial enlargement, and cardiac remodeling (Yoshihisa et al., 2018; Malik et al., 2025). Recent evidence further suggests that, even after adjustment for conventional cardiovascular risk factors, hepatic metabolic abnormalities remain associated with concentric left ventricular remodeling and reduced end-diastolic volume (Saha et al., 2024). These findings indicate that liver fibrosis is not only a marker of MASLD severity, but may also reflect a greater burden of cardiac remodeling. Conversely, chronic inflammation associated with MASLD may alter the myocardial interstitial environment through circulating mediators, whereas low perfusion and venous congestion caused by impaired cardiac function may further aggravate hepatic fibrosis (Sandireddy et al., 2024). Fibrosis in the liver and heart is therefore better understood as a mutually reinforcing process rather than as two independent events.

### 4.5 The gut–Heart–Liver axis and microbial dysbiosis

Gut microbial dysbiosis has gained increasing attention as a complementary mechanism linking MASLD and HFpEF. Under physiological conditions, the gut–liver axis helps maintain metabolic homeostasis and immune balance. In MASLD, however, disruption of the intestinal barrier allows endotoxins such as lipopolysaccharide (LPS) to enter the portal circulation, activate hepatic immune responses, and promote the release of inflammatory and profibrotic mediators, including TNF-α, IL-6, and TGF-β, thereby exacerbating hepatic inflammation and fibrosis (Le Roy et al., 2018; Aron-Wisnewsky et al., 2020; Tripathi et al., 2018).

MASLD is also often accompanied by an altered microbial metabolite profile, characterized by reduced short-chain fatty acids (SCFAs) and increased trimethylamine N-oxide (TMAO) (Boursier et al., 2016; Naghipour et al., 2021). These changes are associated with insulin resistance and hepatic metabolic dysfunction, and may also contribute to endothelial dysfunction, abnormal vascular tone, and myocardial remodeling, thereby potentially increasing the risk of HFpEF (Zhu et al., 2013; Witkowski et al., 2020). In patients with HFpEF, venous congestion and low-grade systemic inflammation may secondarily impair intestinal barrier integrity, increase permeability, and promote the translocation of gut-derived endotoxins into the systemic circulation, thereby triggering myocardial inflammatory cascades and fibrosis (Snelson et al., 2025). The gut–heart–liver axis should therefore be viewed as a sustained amplifier of metabolic imbalance, inflammation, and organ remodeling rather than as an isolated accessory mechanism.

## 5 Clinical identification and risk stratification

### 5.1 From cardiovascular risk to liver-based phenotypic refinement

**FIGURE 3:**
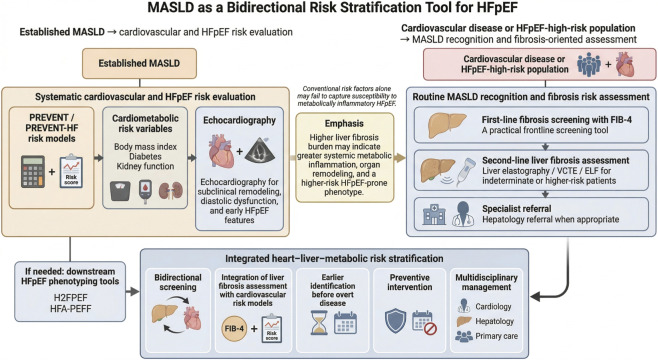
Bidirectional identification and risk stratification of MASLD and HFpEF within a heart–liver–metabolic framework. This figure illustrates a bidirectional clinical framework linking MASLD recognition with cardiovascular and HFpEF risk evaluation, and conversely linking cardiovascular or HFpEF-high-risk phenotypes with fibrosis-oriented liver assessment. The left panel summarizes cardiovascular and HFpEF risk evaluation using PREVENT/PREVENT-HF, cardiometabolic risk profiling, echocardiography, and downstream HFpEF phenotyping tools. The right panel outlines fibrosis-oriented liver assessment using FIB-4 as first-line screening, followed by second-line assessment with liver elastography, vibration-controlled transient elastography (VCTE), or the enhanced liver fibrosis (ELF) test, with hepatology referral when appropriate. The lower panel integrates these pathways into a bidirectional heart–liver–metabolic risk stratification framework for earlier identification, preventive intervention, and multidisciplinary management. Abbreviations: MASLD, metabolic dysfunction–associated steatotic liver disease; HFpEF, heart failure with preserved ejection fraction; PREVENT, Predicting Risk of Cardiovascular Disease Events; PREVENT-HF, heart failure risk prediction model; H2FPEF, Heavy, Hypertensive, Atrial Fibrillation, Pulmonary Hypertension, Elder, Filling Pressure score; HFA-PEFF, Heart Failure Association diagnostic algorithm for heart failure with preserved ejection fraction; FIB-4, Fibrosis-4 index; VCTE, vibration-controlled transient elastography; ELF, enhanced liver fibrosis.

HFpEF is characterized by substantial clinical heterogeneity, and conventional cardiovascular risk factors alone are often insufficient to identify individuals with a greater burden of metabolic inflammation and organ remodeling. In this setting, MASLD should be viewed not only as a common comorbidity, but also as a potential organ-level marker of systemic cardiometabolic stress and susceptibility to HFpEF. In patients with cardiovascular disease or features suggestive of high HFpEF risk, routine recognition of MASLD and fibrosis-oriented liver assessment may therefore help refine phenotyping and risk stratification, as illustrated in Figure 3.

In this setting, FIB-4 may serve as a simple and readily applicable first-line screening tool. It has a high negative predictive value for advanced liver fibrosis and is also associated with adverse cardiovascular outcomes (So-Armah et al., 2017). Using commonly adopted thresholds, a FIB-4 value < 1.3 generally indicates a low likelihood of advanced fibrosis. Values between 1.3 and 2.67 should prompt second-line noninvasive fibrosis assessment, whereas values > 2.67 indicate a higher likelihood of advanced fibrosis and support specialist referral (Rinella et al., 2023b). Because age influences FIB-4 performance, an age-adjusted low-risk threshold should be considered in older adults (Jj et al., 2023). Patients identified as being at intermediate or high risk by FIB-4 should undergo further evaluation with liver elastography or other noninvasive fibrosis assessment tools, with referral to hepatology specialists when appropriate (Chew et al., 2025). From an HFpEF risk stratification perspective, liver fibrosis burden may reflect not only the severity of liver disease, but also a broader burden of systemic metabolic inflammation and organ remodeling. Recognition of MASLD, particularly fibrosis-enriched MASLD, in individuals at high cardiovascular risk may therefore facilitate earlier identification of a metabolically inflammatory subgroup with increased susceptibility to HFpEF.

### 5.2 From MASLD to cardiovascular and HFpEF risk identification

Conversely, in patients with established MASLD, systematic evaluation of cardiovascular disease and heart failure risk is also clinically important. The American Heart Association recently introduced the PREVENT (Predicting Risk of Cardiovascular Disease Events) risk score, which incorporates heart failure as an independent outcome and may provide a pragmatic framework for estimating cardiovascular and heart failure risk in this population (Selvam et al., 2025). Variables included in HF-related risk models, such as body mass index, diabetes, and kidney function, substantially overlap with the cardiometabolic background of MASLD and are broadly consistent with patterns of subclinical cardiac remodeling detected by echocardiography (Mathew et al., 2025). Although these tools were not specifically developed for patients with MASLD, their variable composition suggests that they may help identify individuals who warrant closer cardiac evaluation.

MASLD patients with coexisting obesity, type 2 diabetes, hypertension, chronic kidney disease, or a greater likelihood of liver fibrosis may represent a higher-risk subgroup in whom cardiovascular and heart failure risk should be evaluated more systematically (European Association for the Study of the Liver EASL, 2024a). In these individuals, integration of cardiometabolic risk profiling with echocardiographic assessment may help detect subclinical cardiac remodeling, elevated filling pressures, or other early HFpEF-related features before overt heart failure becomes clinically apparent (Nagueh et al., 2025). Given the high prevalence of MASLD among patients with cardiometabolic HFpEF and the frequent underrecognition of both conditions until later stages, routine identification of MASLD in such individuals may improve overall risk assessment (Miller et al., 2020).

Although recent reviews have proposed an integrated diagnostic approach to cardiometabolic heart–liver disease (Capone et al., 2026), more specific clinical guidance is still lacking on how MASLD-related fibrosis burden should be incorporated into HFpEF-oriented risk enrichment and early identification. C Current heart failure- and MASLD-related guidelines provide limited explicit recommendations regarding bidirectional recognition and coordinated risk stratification. Future efforts to integrate fibrosis assessment with cardiovascular risk models, echocardiographic evaluation, and multidimensional clinical profiling may help identify high-risk individuals earlier and provide a stronger basis for preventive intervention and multidisciplinary management.

## 6 Therapeutic strategies and clinical management

Management of MASLD–HFpEF should extend beyond single-organ approaches to include interventions targeting shared pathophysiological mechanisms. Current strategies mainly include lifestyle intervention and weight reduction, systemic pharmacologic therapies that provide benefits for both heart failure and metabolic dysfunction, and liver-specific disease-modifying therapies represented by resmetirom. In patients with a greater burden of vascular dysfunction, fibrosis, or cardiometabolic comorbidities, adjunctive treatment with nonsteroidal mineralocorticoid receptor antagonists (MRAs), RAAS pathway inhibitors, and other metabolically oriented agents may also be considered. The evidence supporting these therapeutic strategies in HFpEF and MASLD/MASH, as well as their clinical positioning in the context of MASLD–HFpEF overlap, is summarized in Table 2.

**TABLE 2:** Therapeutic strategies for MASLD–HFpEF overlap: integrated evidence and clinical positioning.

Intervention category	Representative interventions	Key evidence in HFpEF	Key evidence in MASLD/MASH	Potential role in MASLD–HFpEF	Key limitations	Ref
Foundational lifestyle intervention	Caloric restriction; exercise training; combined diet–exercise programs; Mediterranean-style dietary optimization	Lifestyle and exercise interventions improve symptoms, functional capacity, and peak oxygen consumption in obesity-related HFpEF. Sustained weight reduction is also associated with favorable hemodynamic and remodeling changes	Sustained weight loss improves steatosis and metabolic dysfunction; greater weight loss may also improve steatohepatitis and fibrosis	Foundational therapy for all MASLD–HFpEF patients; targets shared upstream drivers, including obesity, insulin resistance, inflammation, and vascular load	Long-term adherence and durability remain challenging	European Association for the Study of the Liver EASL (2024b), Jamil et al. (2024), Horn and Tacke (2025)
Bariatric surgery	Roux-en-Y gastric bypass; sleeve gastrectomy; metabolic surgery	Severe-obesity cohorts suggest reverse cardiac remodeling and improvement in diastolic parameters after substantial weight reduction	Bariatric surgery has been associated with improvement in steatosis, steatohepatitis, fibrosis, and multiple metabolic comorbidities	Intensified upstream intervention for selected severe-obesity phenotypes with inadequate response to conservative therapy	Invasive; requires careful patient selection and long-term follow-up	Chauhan et al. (2022), Krishnan et al. (2023), Mummadi et al. (2008)
SGLT2 inhibitors	Empagliflozin; dapagliflozin	EMPEROR-Preserved and DELIVER reduced HF hospitalization or worsening HF. These trials provide the most robust current outcome evidence for SGLT2 inhibitors in HFpEF.	In T2DM-associated MASLD, SGLT2 inhibitors consistently reduce liver fat and aminotransferases; direct histologic benefit on MASH/fibrosis remains less certain	Practical first-line systemic option in overlap patients, especially those with diabetes, CKD, or broad cardiometabolic burden	Direct evidence for MASH resolution or fibrosis regression remains limited; overlap-specific trials are lacking	Anker et al. (2021), Solomon et al. (2022)
GLP-1 receptor agonists	Semaglutide; liraglutide	STEP-HFpEF showed improvement in symptoms, physical limitation, and body weight in obesity-related HFpEF.	In MASH trials, semaglutide increased MASH resolution and reduced liver fat and inflammatory activity, although fibrosis improvement was less consistent	Particularly relevant for obesity-, insulin resistance-, or T2DM-dominant phenotypes; an upstream metabolic strategy with potential benefit across both organ systems	Hard HFpEF outcome data are less mature than for SGLT2 inhibitors; fibrosis reversal remains limited	Newsome et al. (2021), Kosiborod et al. (2023), Kosiborod et al. (2024), Loomba et al. (2023a)
Dual/emerging incretin-based therapies	Tirzepatide; survodutide; efinopegdutide; retatrutide	Emerging data suggest that tirzepatide and related agents may improve symptoms and cardiometabolic burden in obesity-related HFpEF, largely through weight loss and metabolic remodeling	Dual- and multi-agonists show marked reductions in liver fat and promising metabolic and histologic signals in MASLD/MASH-oriented studies	A promising strategy for obesity-dominant and metabolically enriched overlap phenotypes	Direct evidence in dedicated MASLD–HFpEF populations remains limited; long-term outcome data are still evolving	Sanyal et al. (2024a), Romero-Gómez et al. (2023), Author Anonymous (2026), Sanyal et al. (2024b)
Liver-targeted disease-modifying therapy	Resmetirom (THR-β agonist)	No direct HFpEF outcome trial is available; any cardiovascular benefit remains indirect and hypothesis-generating	MAESTRO-NASH established resmetirom as a liver-targeted therapy that improves MASH activity and fibrosis endpoints	Particularly relevant for liver-dominant or fibrosis-enriched phenotypes, especially when hepatic disease burden is a major component of overall risk	No proven direct benefit on HFpEF symptoms, cardiac structure, or exercise capacity	Harrison et al. (2024), Kingwell (2024)
Fibrosis-/cardiorenal-oriented adjunctive therapy	Finerenone	FINEARTS-HF supports benefit on worsening HF/CV events in relevant EF ranges, with strong cardiorenal-metabolic and antifibrotic rationale	Direct liver-specific outcome data remain limited, although finerenone remains mechanistically attractive because of its anti-inflammatory and antifibrotic profile	Adjunctive phenotype-oriented option for patients with CKD, hypertension, diabetes, or increased fibrosis/remodeling burden	Limited direct evidence in MASLD–HFpEF overlap; not a liver-specific disease-modifying therapy	DeFronzo and Bakris (2022), Heinig and Eissing (2023), Wang et al. (2025), Ismahel and Docherty (2025)
RAAS pathway modulation	ACE inhibitors; ARBs; ARNI	HFpEF benefit is background- or phenotype-specific and generally more modest than in HFrEF, although selected subgroups may derive symptomatic or remodeling benefit	Possible indirect effects on metabolic and fibrotic pathways have been suggested, but robust MASLD-specific clinical outcome data are lacking	Supplementary option for selected hypertension-, afterload-, or remodeling-dominant phenotypes	Overlap-specific evidence is limited; benefits are mainly indirect in the MASLD–HFpEF context	Ponikowski et al. (2016), Martin et al. (2021), Yoon and Eom (2019), Solomon et al. (2019), McMurray et al. (2014)
Other comorbidity-directed therapies	Statins; metformin; pioglitazone	Statins mainly reduce ASCVD risk; metformin provides indirect cardiometabolic benefit; pioglitazone is constrained by edema and HF-related safety concerns	Statins are safe in MASLD; metformin has limited histologic efficacy; pioglitazone may improve MASH in selected patients	Useful for broader comorbidity control, but not core overlap-specific therapies	Benefits are mostly indirect; pioglitazone may cause weight gain and fluid retention	Ali et al. (2009), Haramaki et al. (2007), Aoki et al. (1997), Bailey (2024), Stahl et al. (2019), Allocca et al. (2025), Boettcher et al. (2012), Francque et al. (2021a), Barb et al. (2016), Musso et al. (2017), Lebovitz (2019)

Evidence across rows is heterogeneous and includes hard HFpEF, outcomes, symptom/functional improvements, liver histologic endpoints, metabolic surrogate changes, and mechanistic rationale; direct evidence in dedicated MASLD–HFpEF, overlap populations remains limited for most interventions.

### 6.1 Lifestyle intervention and weight management

Lifestyle intervention is the foundation of management for both MASLD and HFpEF and underpins other therapeutic strategies. Dietary modification, exercise training, and weight reduction can improve blood pressure, vascular function, and insulin sensitivity, while also reducing hepatic fat accumulation and overall cardiometabolic burden (Lassailly et al., 2015; El Meouchy et al., 2022; Swift et al., 2018; Chandra et al., 2014).

In MASLD, sustained weight loss can improve hepatic insulin resistance, steatosis, and inflammatory activity (Petersen et al., 2005). When weight reduction is substantial and maintained over time, fibrosis may also improve (You et al., 2012; Vilar-Gomez et al., 2015). In HFpEF, obesity is closely linked to disease risk, whereas weight loss may reduce hemodynamic burden, improve inflammatory status, and enhance exercise capacity (Borlaug et al., 2010; Reddy et al., 2019).

Among dietary interventions, the Mediterranean diet is the most representative and widely recommended pattern for MASLD management. The latest joint guidelines from EASL, EASD, and EASO identify it as an important component of lifestyle intervention because of its favorable effects on hepatic metabolism and inflammation (European Association for the Study of the Liver EASL, 2024b; Jamil et al., 2024; Horn and Tacke, 2025). Plant-based dietary patterns have also been associated with better glycemic control, improved blood pressure, and a lower risk of heart failure (Micha et al., 2017; Lara et al., 2019). The DASH (Dietary Approaches to Stop Hypertension) diet is well supported for blood pressure control and has also been associated with improved diastolic and arterial function (Levitan et al., 2013). In patients with MASLD, it has shown potential to improve steatosis, liver enzyme levels, and fibrosis-related indices (Sangouni et al., 2024; Rees et al., 2019). Thus, dietary optimization may influence not only liver-related outcomes, but also the metabolic and hemodynamic burden relevant to HFpEF.

Combined diet and exercise interventions are generally more effective than either strategy alone. A recent meta-analysis showed that, in patients with MASLD, comprehensive lifestyle intervention was more effective than single-component strategies in improving liver enzyme levels and insulin sensitivity (Fernández et al., 2022). For patients with severe obesity who are unable to achieve meaningful weight loss through lifestyle measures alone, bariatric surgery may be considered as an intensified treatment option. Studies have shown that bariatric surgery can improve steatosis, inflammation, and fibrosis in MASLD, while also ameliorating cardiometabolic comorbidities such as type 2 diabetes, hypertension, and dyslipidemia (Chauhan et al., 2022; Krishnan et al., 2023; Mummadi et al., 2008). Substantial weight loss has also been associated with reverse cardiac remodeling, reductions in left atrial size and left ventricular mass index, and improved diastolic function (Lascaris et al., 2018; Sorimachi et al., 2022).

Lifestyle intervention should therefore be regarded as a foundational component of MASLD–HFpEF management rather than a purely adjunctive measure. Its principal value lies in targeting several key pathways simultaneously, including excess body weight, insulin resistance, inflammation, and hemodynamic load.

### 6.2 SGLT2 inhibitors

SGLT2 inhibitors currently represent one of the most clinically feasible systemic therapeutic options for MASLD–HFpEF overlap. Although these agents were initially developed as glucose-lowering drugs, their benefits in heart failure have since been established in multiple cardiovascular outcome trials (Brown et al., 2021a). Beyond reducing volume overload and improving glycemic control, SGLT2 inhibitors may also influence HFpEF pathophysiology through several mechanisms, including improved myocardial energy utilization, attenuation of inflammation- and fibrosis-related responses, and improvement of coronary microvascular endothelial function (Mathew et al., 2025; Delanaye and Scheen, 2021).

Two landmark trials have established the role of SGLT2 inhibitors in HFpEF. In EMPEROR-Preserved, empagliflozin reduced the risk of cardiovascular death or first hospitalization for heart failure (Anker et al., 2021). DELIVER subsequently showed a similar reduction in cardiovascular death or worsening heart failure with dapagliflozin (Solomon et al., 2022). SGLT2 inhibitors are therefore among the best-supported therapies currently available for improving clinical outcomes in HFpEF.

On the hepatic side, the potential benefits of SGLT2 inhibitors in MASLD were first observed mainly in patients with coexisting type 2 diabetes. Randomized studies and meta-analyses have shown that these agents reduce liver fat content and improve liver enzyme levels (Shi et al., 2023), p potentially through suppression of *de novo* lipogenesis, enhancement of fatty acid β-oxidation, and improvement of insulin resistance (Zhang and Hu, 2026; Lin et al., 2024; Ma et al., 2022). However, clinical evidence supporting histologic resolution of MASH or direct antifibrotic effects remains limited.

Overall, the value of SGLT2 inhibitors lies in their ability to address both heart failure outcomes and broader metabolic dysfunction. The 2022 AHA/ACC/HFSA heart failure guideline assigned SGLT2 inhibitors a Class IIa recommendation for HFpEF (Heidenreich et al., 2022), and current evidence further suggests that they may improve quality of life and exercise capacity (Treewaree et al., 2023; Jaiswal et al., 2023; Gao et al., 2024). In addition, combination therapy with SGLT2 inhibitors and GLP-1 receptor agonists may provide additive benefits in body weight, glycemic control, and blood pressure, offering a potential strategy for integrated metabolic intervention in MASLD-related HFpEF (Kunutsor et al., 2024; Zhou et al., 2020). In patients with HFpEF and coexisting MASLD-related metabolic abnormalities, SGLT2 inhibitors therefore represent one of the systemic treatment options with the strongest current clinical support.

### 6.3 Incretin-based therapies

Therapies targeting the incretin pathway are increasingly being incorporated into the treatment of metabolic disease. Whether in the form of single-target GLP-1 receptor agonists, dual GLP-1/GIP receptor agonists, or earlier-stage triple agonists, these agents share the ability to act on upstream drivers such as obesity, insulin resistance, visceral adiposity, and chronic low-grade inflammation (Drucker, 2022). These properties have generated considerable interest in their potential role in MASLD and HFpEF (Newsome et al., 2021; Butler et al., 2023).

Among the currently available agents, GLP-1 receptor agonists are the most extensively studied. In STEP-HFpEF, semaglutide 2.4 mg significantly improved symptoms, physical limitations, and exercise function in patients with obesity-related HFpEF, while also producing substantial weight loss (Kosiborod et al., 2023). STEP-HFpEF DM further showed that these benefits extended to obese HFpEF patients with coexisting type 2 diabetes (Kosiborod et al., 2024). On the hepatic side, semaglutide has been shown to increase the rate of MASH resolution, although its effect on fibrosis improvement appears more limited (Newsome et al., 2021; Loomba et al., 2023a).

Dual receptor agonists further expand this therapeutic pathway. In the SUMMIT trial, tirzepatide reduced the composite risk of cardiovascular death or worsening heart failure in patients with obesity-related HFpEF and also improved health status (Packer et al., 2025). Dual incretin-based agents such as tirzepatide, survodutide, and efinopegdutide have also shown promising effects on MASH-related metabolic abnormalities, hepatic fat content, and histologic resolution (Sanyal et al., 2024a; Romero-Gómez et al., 2023; Author Anonymous, 2026). BBy contrast, triple receptor agonists remain at an earlier stage of development. Although retatrutide has shown marked reductions in liver fat content together with greater weight-loss effects, direct clinical evidence in patients with coexisting MASLD and HFpEF remains limited (Sanyal et al., 2024b).

Overall, incretin-based therapies represent a more mechanism-oriented approach to MASLD–HFpEF management by targeting shared metabolic drivers across organs. For patients characterized predominantly by obesity, insulin resistance, and visceral adiposity, these agents may offer a relevant upstream therapeutic option. Nevertheless, their long-term benefits, optimal target population, and precise positioning within MASLD–HFpEF management remain to be further defined (Author Anonymous, 2026; Sanyal et al., 2024b).

### 6.4 Combination therapeutic strategies

Combination strategies may be particularly relevant in MASLD–HFpEF overlap, where obesity, type 2 diabetes, chronic kidney disease, and fibrosis-related metabolic risk frequently coexist. Among currently available options, combined treatment with SGLT2 inhibitors and GLP-1 receptor agonists is of particular interest because these agents act on complementary components of cardiometabolic disease biology (Brown et al., 2021b). SGLT2 inhibitors are supported primarily by their effects on heart failure outcomes and cardiorenal protection, whereas GLP-1 receptor agonists more directly target upstream metabolic drivers such as obesity, insulin resistance, and adiposity-related inflammation (Kosiborod et al., 2023; Kittleson et al., 2023). In real-world analyses, combined GLP-1 receptor agonist and SGLT2 inhibitor therapy has been associated with an approximately 30% lower risk of major adverse cardiovascular events than GLP-1 receptor agonist or SGLT2 inhibitor monotherapy, with a parallel signal for reduced serious renal events, although renal estimates were less precise in some comparisons (Simms-Williams et al., 2024). A broader synthesis of observational studies also suggested greater reductions in major adverse cardiovascular events, heart failure hospitalization, and all-cause mortality with combination treatment than with either drug class alone (Scheen, 2025). In obesity- or diabetes-dominant MASLD–HFpEF phenotypes, these findings support consideration of combined therapy as a broader cardiometabolic strategy than either agent alone.

However, the evidence remains indirect. Available data are derived largely from real-world studies and from populations enriched for obesity, diabetes, or broader cardiometabolic risk rather than from dedicated MASLD–HFpEF trials. Important questions therefore remain regarding patient selection, treatment sequencing, tolerability, cost-effectiveness, and the extent to which combination regimens can improve liver-related and HFpEF-related outcomes in parallel (Kittleson et al., 2023; Zhou et al., 2026). For now, combination therapy is best regarded as a clinically relevant but still evolving option for selected metabolically enriched phenotypes.

### 6.5 THR-β agonists

Resmetirom is a liver-selective thyroid hormone receptor beta (THR-β) agonist that may slow fibrosis progression by promoting hepatic lipid metabolism, reducing intrahepatic fat accumulation, and alleviating lipotoxicity (Younossi et al., 2025b). Unlike SGLT2 inhibitors or incretin-based therapies, resmetirom is distinguished by its direct effects on hepatic metabolic abnormalities and its disease-modifying potential for liver-related endpoints (Harrison et al., 2024).

In the phase 3 MAESTRO-NASH trial, resmetirom significantly increased the rate of NASH resolution and improved fibrosis outcomes in patients with NASH and coexisting liver fibrosis (Harrison et al., 2024). The 2024 U.S. Food and Drug Administration approval of resmetirom for noncirrhotic NASH with F2–F3 fibrosis marked an important step toward the clinical use of disease-modifying treatment (Kingwell, 2024).

In patients with coexisting MASLD and HFpEF, the potential value of resmetirom lies primarily in its ability to improve hepatic lipid metabolism and slow fibrotic progression, thereby indirectly reducing systemic metabolically inflammatory burden and cardiovascular risk (Mustafa et al., 2025). However, current evidence remains focused mainly on hepatic endpoints, and dedicated data in MASLD–HFpEF overlap populations are still lacking. Resmetirom may be most relevant in patients with greater hepatic disease burden or increased fibrosis risk, although its role in MASLD–HFpEF overlap remains to be more clearly defined.

### 6.6 Nonsteroidal MRAs and RAAS pathway–targeted therapies

Finerenone is a novel nonsteroidal mineralocorticoid receptor antagonist (MRA) with greater receptor selectivity than conventional spironolactone and a lower propensity for hormone-related adverse effects (DeFronzo and Bakris, 2022; Heinig and Eissing, 2023). In patients with diabetic kidney disease, the FIDELIO-DKD and FIGARO-DKD trials showed that finerenone reduces cardiovascular events and adverse renal outcomes in those with type 2 diabetes and chronic kidney disease, providing both mechanistic and clinical support for its evaluation in HFpEF (164). The FINEARTS-HF trial further showed that finerenone reduced the composite risk of worsening heart failure events and cardiovascular death in patients with HFmrEF and HFpEF. These findings suggest that nonsteroidal MRAs may offer an additional therapeutic option in HFpEF beyond SGLT2 inhibitors, potentially through anti-inflammatory, antifibrotic, and cardiorenal-metabolic effects. Nevertheless, dedicated evidence in patients with coexisting MASLD and HFpEF remains limited, and finerenone is currently better viewed as a mechanism-based, phenotype-oriented adjunctive therapy (Ismahel and Docherty, 2025).

ACE inhibitors (ACEIs), angiotensin receptor blockers (ARBs), and angiotensin receptor–neprilysin inhibitors (ARNIs) are best regarded as background or subgroup-directed therapies in HFpEF. ACEIs and ARBs reduce blood pressure, lower afterload, and attenuate neurohormonal activation through RAAS inhibition. Although their benefits are well established in HFrEF, their effects on major clinical endpoints in HFpEF appear more modest (Ponikowski et al., 2016; Martin et al., 2021; Yoon and Eom, 2019). From the perspective of MASLD, RAAS inhibition may confer some metabolic benefit by improving insulin resistance and attenuating inflammation- and fibrosis-related processes, although clinical evidence for meaningful translational benefit remains limited (Li et al., 2022; Li et al., 2018; Thethi et al., 2012). For ARNIs, PARAGON-HF did not meet its primary endpoint in the overall study population, although signals of reduced hospitalization risk were observed in selected subgroups (Solomon et al., 2019; McMurray et al., 2014). The value of these agents in MASLD–HFpEF therefore lies mainly in their supplementary effects on vascular tone, neurohormonal activation, inflammation, and fibrotic remodeling, particularly in patients with coexisting hypertension, renal dysfunction, or a greater fibrosis burden (Kittleson et al., 2023; Desai et al., 2023; Kitai et al., 2025).

### 6.7 Other metabolically relevant therapies

Conventional metabolic agents, such as statins, metformin, and thiazolidinediones, are used primarily to manage cardiometabolic comorbidities in patients with MASLD, including dyslipidemia, type 2 diabetes mellitus, and insulin resistance, and may provide additional hepatic and cardiovascular benefits (Zisis et al., 2025). However, these agents were not specifically developed for MASLD or HFpEF. In the context of MASLD–HFpEF overlap, their role is therefore better viewed as one of comorbidity control and risk modification rather than core disease-modifying therapy.

Statins remain a cornerstone of primary and secondary cardiovascular prevention, and their main value in MASLD lies in reducing atherosclerotic cardiovascular risk rather than serving as a specific therapy for MASLD or HFpEF (177–179). Metformin, a first-line therapy for type 2 diabetes, has not shown consistent histologic benefit in MASLD, although it may still exert modest effects on body weight, liver enzyme levels, and insulin resistance (Bailey, 2024; Stahl et al., 2019; Allocca et al., 2025). At present, there is insufficient evidence to support its use as a specific treatment for MASLD or HFpEF, and its clinical role therefore remains largely confined to the management of dysglycemia and related metabolic comorbidities (Huang et al., 2022).

Thiazolidinediones such as pioglitazone improve insulin sensitivity, regulate glucose metabolism, and attenuate inflammatory responses through activation of peroxisome proliferator-activated receptor gamma (PPARγ), and have shown some therapeutic potential in patients with MASH(184–187). However, because they may cause weight gain and fluid retention, their use in patients with heart failure is substantially limited (Lebovitz, 2019). In the setting of MASLD–HFpEF overlap, these agents are better regarded as adjunctive options for selected comorbidity management than as preferred therapeutic choices.

### 6.8 Emerging therapies and Future directions

As understanding of MASLD and its associated comorbidities continues to evolve, multiple targeted therapies are being developed, particularly those aimed at anti-inflammatory, antifibrotic, and hepatic metabolic remodeling pathways. For example, lanifibranor, pegozafermin, and other fibroblast growth factor 21 (FGF21) analogs have shown promising signals for improving MASH activity, reducing liver fat content, and promoting fibrosis regression (Francque S. M. et al., 2021; Sanyal et al., 2019; Loomba et al., 2023b; Harrison et al., 2023). Investigational agents targeting acetyl-CoA carboxylase (ACC), diacylglycerol acyltransferase 2 (DGAT2), and galectin-3 have also shown potential to improve hepatic histologic features (Zetterberg et al., 2022; Calle et al., 2021). Nonetheless, these therapies remain focused primarily on liver-specific endpoints, and robust evidence regarding their direct effects on HFpEF symptoms, structural remodeling, and exercise capacity is still lacking.

Overall, treatment of MASLD–HFpEF is moving toward strategies that target shared disease mechanisms across organs. At present, the most evidence-supported approach centers on lifestyle intervention, SGLT2 inhibitors, and upstream metabolic therapies based on incretin pathways. Resmetirom may be particularly relevant in patients with greater hepatic disease burden or increased fibrosis risk, whereas nonsteroidal MRAs, RAAS pathway inhibitors, and other metabolic agents are better regarded as adjunctive therapies for selected phenotypes or comorbidity-driven management. A likely future direction lies in the rational integration of liver-directed therapies with SGLT2 inhibitors, incretin-based therapies, and other cardioprotective strategies to address liver disease progression, metabolic burden, and heart failure outcomes in a coordinated manner.

## 7 Conclusion

MASLD and HFpEF are increasingly encountered within the same cardiometabolic setting, and current evidence suggests that their relationship is more than incidental coexistence. Epidemiological studies support an association between MASLD and incident heart failure, with emerging evidence suggesting particular relevance to the HFpEF phenotype. At the mechanistic level, insulin resistance, lipotoxicity, chronic low-grade inflammation, microvascular dysfunction, neurohormonal activation, and fibrosis provide a plausible biological basis for this overlap, suggesting that both conditions may arise from a shared metabolically inflammatory milieu rather than from independent organ-specific processes.

These observations also carry important clinical implications. In particular, MASLD accompanied by a greater fibrosis burden may help identify patients with more advanced metabolic derangement and a higher likelihood of adverse cardiac remodeling. From this perspective, liver fibrosis assessment may contribute to cardiovascular risk refinement, while structured cardiac evaluation may facilitate earlier identification of high-risk individuals within the MASLD population.

Management is likewise evolving. Lifestyle intervention remains fundamental, and newer agents such as SGLT2 inhibitors, incretin-based therapies, and selected liver-directed treatments provide a broader therapeutic framework for this overlap phenotype. Even so, direct prospective evidence in patients with coexisting MASLD and HFpEF remains limited.

Future research should move beyond descriptive association. Prospective studies need to define clinically meaningful MASLD–HFpEF phenotypes, particularly those characterized by obesity, type 2 diabetes, visceral adiposity, or greater fibrosis burden. Risk stratification pathways that combine fibrosis-oriented liver assessment with echocardiography and HFpEF-focused clinical evaluation also require formal validation in real-world populations. In parallel, interventional studies should determine whether therapies targeting shared metabolic and fibrotic pathways can improve liver-related and HFpEF-related outcomes in tandem. Addressing these questions will be essential for translating the concept of MASLD–HFpEF overlap into a more practical and integrated management strategy.
